# Poly[[μ-1,4-bis­(pyridin-4-ylmeth­yl)piperazine]bis­[μ_3_-4-(2-carboxyl­atoeth­yl)benzoato]dicopper(II)]

**DOI:** 10.1107/S2414314623007459

**Published:** 2023-08-30

**Authors:** Jason Jia, Robert L. LaDuca

**Affiliations:** a E-194 Holmes Hall, Michigan State University, Lyman Briggs College, 919 E. Shaw Lane, East Lansing, MI 48825, USA; Vienna University of Technology, Austria

**Keywords:** crystal structure, copper(II), Jahn–Teller distortion, coordination polymer

## Abstract

In the title coordination polymer, [Cu_2_(C_9_H_6_O_4_)_2_(C_16_H_20_N_4_)]_*n*_, the Cu^II^ atoms in {NO_4_} square-pyramidal coordination environments are conjoined into diperiodic coordination polymer slabs by the full span of the bridging 1,4-bis­(pyridin-4-ylmeth­yl)piperazine (bpmp) and 4-(carboxyl­eth­yl)benzoate (ceb) ligands. The slab motifs are expanded into the full crystal structure by means of longer-range C—H⋯O attractive inter­actions.

## Abstract 2

The title com­pound is a diperiodic slab copper(II) coordination polymer held together *via* longer-range C—H⋯O attractive inter­actions.

## Graphical abstract

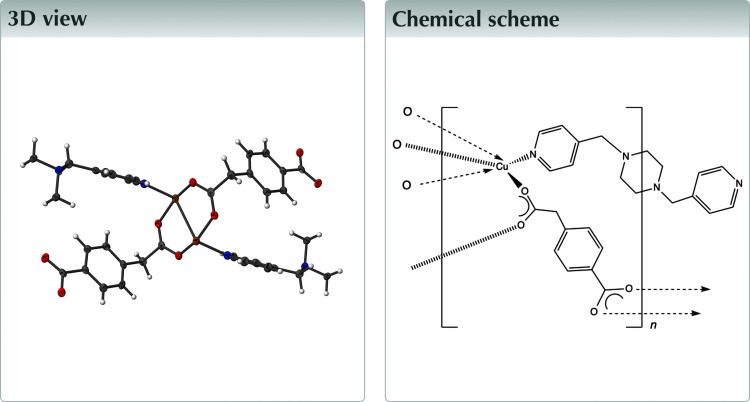

## Structure description

Our group has reported several divalent metal coordination polymers with intriguing topologies based on the dipodal pyridyl ligand 1,4-bis­(pyridin-4-ylmeth­yl)piperazine (bpmp) in the presence of di­carboxyl­ate co-ligands. For example, using the di­carboxyl­ate ligand oxy(bis­benzoate) (oba) afforded the highly entangled self-penetrated phase [Co_3_(oba)_3_(bpmp)_2_] (Martin *et al.*, 2008 ▸). The title com­pound was obtained by hydro­thermal reaction of copper nitrate, 4-(carboxyl­eth­yl)benzoic acid (cebH_2_), and bpmp under basic conditions.

**Figure 1:**
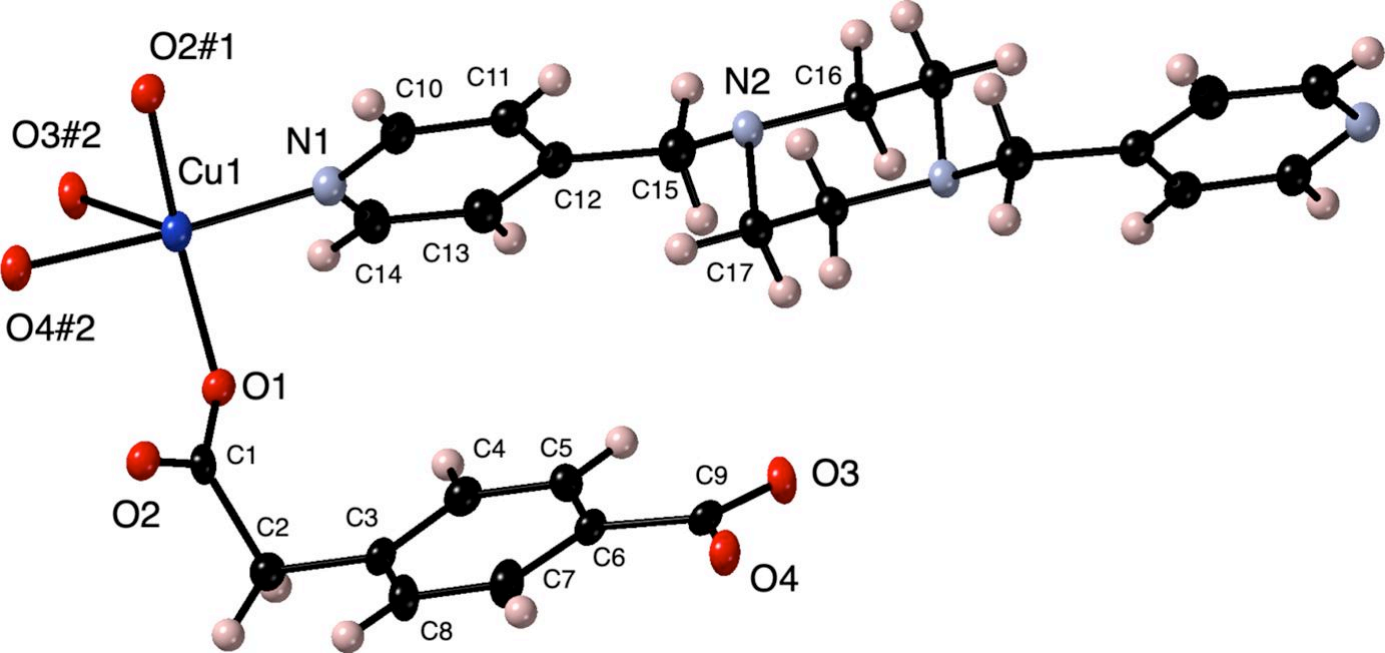
The copper coordination environment in the title com­pound with full ceb and bpmp ligands. Displacement ellipsoids are drawn at the 50% probability level. Color code: Cu dark blue, O red, N light blue, C black, and H pink. The symmetry codes are as listed in Table 1 ▸.

The asymmetric unit of the title com­pound consists of a Cu^II^ atom, a ceb ligand, and half of a bpmp ligand whose central piperazine ring is situated on a crystallographic inversion center. The Cu^II^ atom is coordinated in a {NO_4_} square-pyramidal arrangement (Fig. 1 ▸) with ‘longer’ arm ceb carboxyl­ate O-atom donors in *trans* positions in the basal plane. A carboxyl­ate group from the ‘shorter’ arm ceb terminus bridges a basal position and the Jahn–Teller elongated apical position. The remaining coordination site in the basal plane is taken up by a pyridyl N-atom donor from a bpmp ligand. A modest deviation from idealized square-pyramidal coordination is indicated by the trigonality factor τ of 0.11 (Addison *et al.*, 1984 ▸). Bond lengths and angles within the coordination sphere are listed in Table 1 ▸.

**Figure 2:**
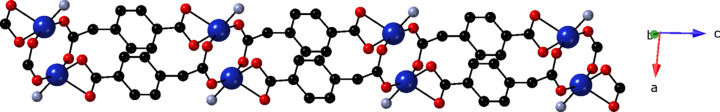
The [Cu_2_(ceb)_2_]_*n*_ coordination polymer chain in the title com­pound.

**Figure 3:**
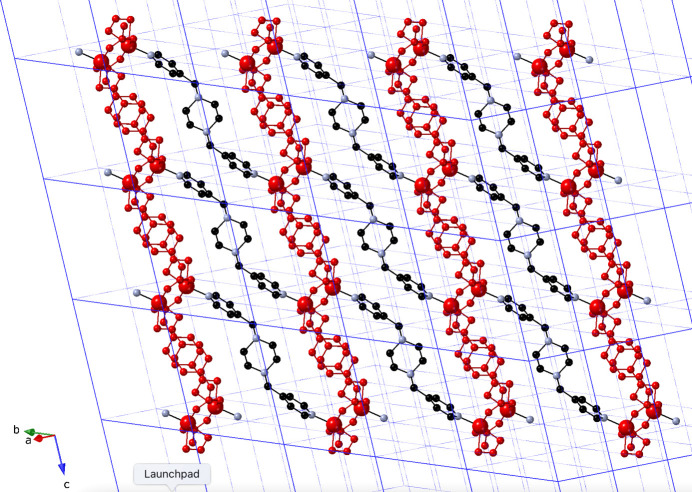
The [Cu_2_(ceb)_2_(bpmp)]_*n*_ coordination polymer slab in the title com­pound. The [Cu_2_(ceb)_2_]_*n*_ chain motifs are drawn in red.

**Figure 4:**
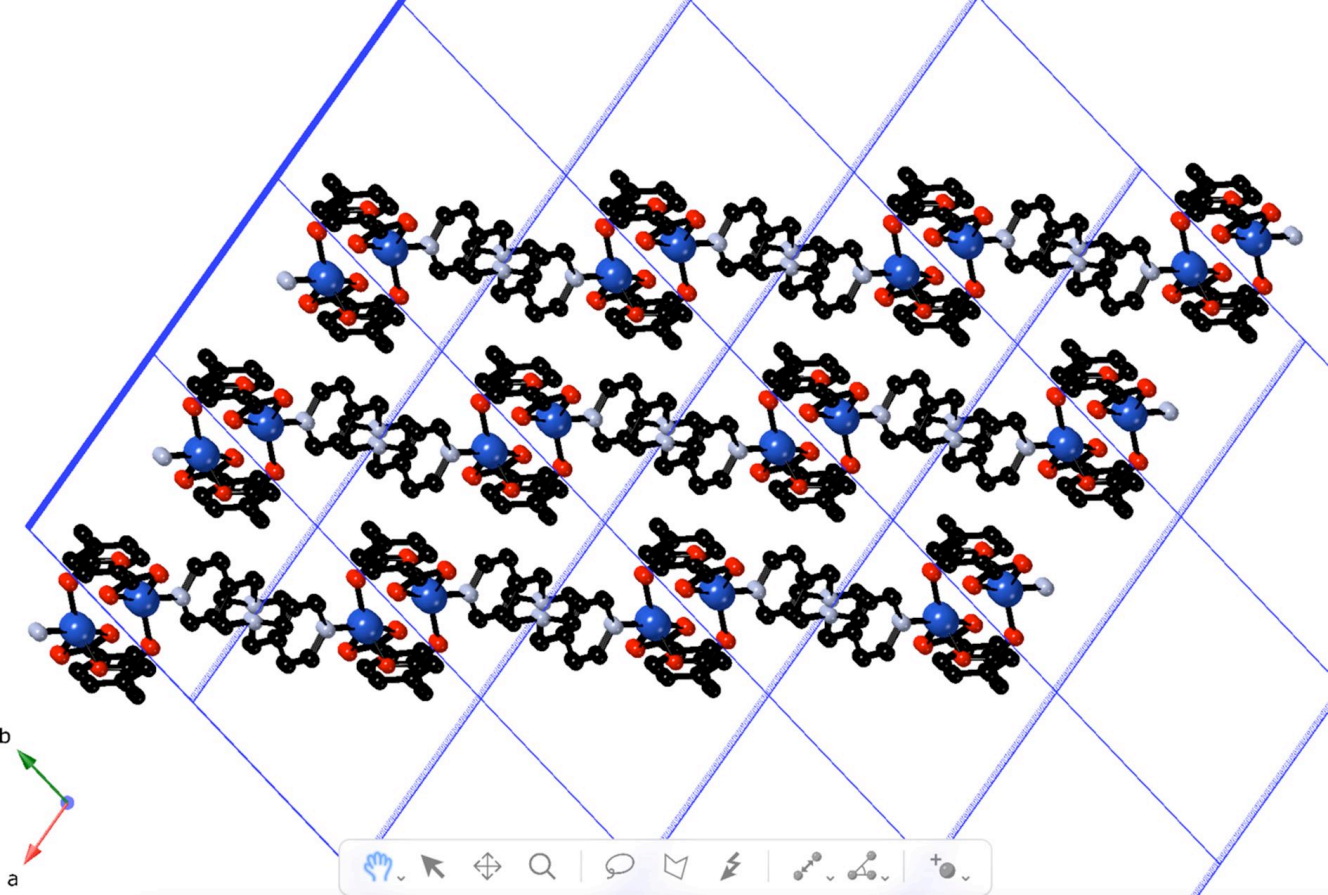
The *AAA* stacking of the [Cu_2_(ceb)_2_(bpmp)]_*n*_ coordination polymer slabs in the title com­pound.

The carboxyl­ate groups of the longer arms of the ceb ligands bridge two Cu^II^ atoms in a *syn*–*syn* fashion to construct [Cu_2_(OCO)_2_] dimeric groups with a Cu⋯Cu distance of 2.8992 (8) Å. These are connected by chelating carboxyl­ate groups belonging to the shorter ceb termini, to form [Cu_2_(ceb)_2_] monoperiodic coordination polymer ribbons oriented along the *c* axis (Fig. 2 ▸). These [Cu_2_(ceb)_2_] ribbon motifs are pillared by dipodal bpmp ligands to form [Cu_2_(ceb)_2_(bpmp)]_*n*_ coordination polymer slabs that are oriented parallel to (1 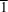 0) (Fig. 3 ▸). Longer-range C—H⋯O attractive forces between parallel adjacent slab motifs construct the full three-dimensional crystal structure of the title com­pound (Fig. 4 ▸). The slabs stack in an *AAA* repeating pattern along the *a* crystal direction.

## Synthesis and crystallization

Cu(NO_3_)_2_·2.5H_2_O (86 mg, 0.37 mmol), 4-(carb­oxy­meth­yl)benzoic acid (cmbH_2_) (67 mg, 0.37 mmol), 1,4-bis­(pyridin-4-ylmeth­yl)piperazine (bpmp) (99 mg, 0.37 mmol), and 0.75 ml of a 1.0 *M* NaOH solution were placed in 10 ml distilled water in a Teflon-lined acid digestion bomb. The bomb was sealed and heated in an oven at 393 K for 48 h, and then cooled slowly to 273 K. Green crystals of the title com­plex were obtained in 51% yield.

## Refinement

Crystal data, data collection and structure refinement details are summarized in Table 2 ▸. The greatest remaining electron density of 1.53 e Å^−3^ is situated 1.45 Å from the Cu1 atom.

## Supplementary Material

Crystal structure: contains datablock(s) I, 1R. DOI: 10.1107/S2414314623007459/wm4195sup1.cif

Structure factors: contains datablock(s) I. DOI: 10.1107/S2414314623007459/wm4195Isup2.hkl

res file. DOI: 10.1107/S2414314623007459/wm4195sup3.txt

CCDC reference: 1976250

Additional supporting information:  crystallographic information; 3D view; checkCIF report

## Figures and Tables

**Table 1:** Selected geometric parameters (Å, °)

Cu1—O1	1.969 (2)	Cu1—O4^ii^	1.985 (2)
Cu1—O2^i^	1.968 (2)	Cu1—N1	1.979 (3)
Cu1—O3^ii^	2.299 (2)		
			
O1—Cu1—O3^ii^	101.92 (9)	O2^i^—Cu1—O4^ii^	90.56 (10)
O1—Cu1—O4^ii^	92.30 (10)	O2^i^—Cu1—N1	93.02 (11)
O1—Cu1—N1	89.47 (11)	O4^ii^—Cu1—O3^ii^	61.65 (9)
O2^i^—Cu1—O1	157.53 (10)	N1—Cu1—O3^ii^	104.62 (10)
O2^i^—Cu1—O3^ii^	99.04 (9)	N1—Cu1—O4^ii^	166.22 (11)

Symmetry codes: (i) 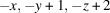 ; (ii) 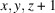 .

**Table 2:** Experimental details

Crystal data	
Chemical formula	[Cu_2_(C_9_H_6_O_4_)_2_(C_16_H_20_N_4_)]
*M*_r_	751.71
Crystal system, space group	Triclinic, *P* 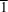
Temperature (K)	173
*a*, *b*, *c* (Å)	8.5431 (8), 9.7391 (9), 9.9667 (9)
α, β, γ (°)	104.523 (1), 93.049 (1), 99.966 (1)
*V* (Å^3^)	786.57 (13)
*Z*	1
Radiation type	Mo *K*α
μ (mm^−1^)	1.41
Crystal size (mm)	0.20 × 0.11 × 0.07

Data collection	
Diffractometer	Bruker APEXII CCD
Absorption correction	Multi-scan (*SADABS*; Bruker, 2014 ▸)
*T*_min_, *T*_max_	0.693, 0.745
No. of measured, independent and observed [*I* > 2σ(*I*)] reflections	10964, 2883, 2459
*R*_int_	0.042
(sin θ/λ)_max_ (Å^−1^)	0.602

Refinement	
*R*[*F* ^2^ > 2σ(*F* ^2^)], *wR*(*F* ^2^), *S*	0.044, 0.114, 1.12
No. of reflections	2883
No. of parameters	217
H-atom treatment	H-atom parameters constrained
Δρ_max_, Δρ_min_ (e Å^−3^)	1.53, −0.32

Computer programs: *COSMO* (Bruker 2009 ▸), *SAINT* (Bruker, 2014 ▸), *SHELXT* (Sheldrick, 2015*a* ▸), *SHELXL* (Sheldrick, 2015*b* ▸), *CrystalMakerX* (Palmer, 2020 ▸), and *OLEX2* (Dolomanov *et al.*, 2009 ▸).

## full crystallographic data

### Crystal data

**Table:**

[Cu_2_(C_9_H_6_O_4_)_2_(C_16_H_20_N_4_)]	*Z* = 1
*M**_r_* = 751.71	*F*(000) = 386
Triclinic, *P*1	*D*_x_ = 1.587 Mg m^−^^3^
*a* = 8.5431 (8) Å	Mo *K*α radiation, λ = 0.71073 Å
*b* = 9.7391 (9) Å	Cell parameters from 5378 reflections
*c* = 9.9667 (9) Å	θ = 2.2–25.3°
α = 104.523 (1)°	µ = 1.41 mm^−^^1^
β = 93.049 (1)°	*T* = 173 K
γ = 99.966 (1)°	Plate, green
*V* = 786.57 (13) Å^3^	0.20 × 0.11 × 0.07 mm

### Data collection

**Table:**

Bruker APEXII CCD diffractometer	2883 independent reflections
Radiation source: sealed tube	2459 reflections with *I* > 2σ(*I*)
Graphite monochromator	*R*_int_ = 0.042
Detector resolution: 8.36 pixels mm^-1^	θ_max_ = 25.3°, θ_min_ = 2.1°
ω scans	*h* = −10→10
Absorption correction: multi-scan (SADABS; Bruker, 2014)	*k* = −11→11
*T*_min_ = 0.693, *T*_max_ = 0.745	*l* = −11→11
10964 measured reflections	

### Refinement

**Table:**

Refinement on *F*^2^	Primary atom site location: dual
Least-squares matrix: full	Hydrogen site location: inferred from neighbouring sites
*R*[*F*^2^ > 2σ(*F*^2^)] = 0.044	H-atom parameters constrained
*wR*(*F*^2^) = 0.114	*w* = 1/[σ^2^(*F*_o_^2^) + (0.0559*P*)^2^ + 0.6812*P*] where *P* = (*F*_o_^2^ + 2*F*_c_^2^)/3
*S* = 1.12	(Δ/σ)_max_ = 0.001
2883 reflections	Δρ_max_ = 1.53 e Å^−^^3^
217 parameters	Δρ_min_ = −0.31 e Å^−^^3^
0 restraints	

### Special details

**Table:**

Experimental. Data was collected using a BRUKER CCD (charge coupled device) based diffractometer equipped with an Oxford low-temperature apparatus operating at 173 K. A suitable crystal was chosen and mounted on a nylon loop using Paratone oil. Data were measured using omega scans of 0.5° per frame for 30 s. The total number of images were based on results from the program COSMO where redundancy was expected to be 4 and completeness to 0.83Å to 100%. Cell parameters were retrieved using APEX II software and refined using SAINT on all observed reflections.Data reduction was performed using the SAINT software which corrects for Lp. Scaling and absorption corrections were applied using SADABS6 multi-scan technique, supplied by George Sheldrick. The structure was solved by the direct method using the SHELXT program and refined by least squares method on F2, SHELXL, incorporated in OLEX2.
Geometry. All esds (except the esd in the dihedral angle between two l.s. planes) are estimated using the full covariance matrix. The cell esds are taken into account individually in the estimation of esds in distances, angles and torsion angles; correlations between esds in cell parameters are only used when they are defined by crystal symmetry. An approximate (isotropic) treatment of cell esds is used for estimating esds involving l.s. planes.
Refinement. The structure was refined by Least Squares using version 2018/3 of XL (Sheldrick, 2015b) incorporated in Olex2 (Dolomanov *et al.*, 2009). All non-hydrogen atoms were refined anisotropically. Hydrogen atom positions were calculated geometrically and refined using the riding model, except for the Hydrogen atom on the nitrogen atom which was found by difference Fourier methods and refined isotropically. There is an unresolvable absorption artifact located as a difference peak of 1.53 e^-^ Å^-3^ situated 1.45 Å from the Cu1 atom.

### Fractional atomic coordinates and isotropic or equivalent isotropic displacement parameters (Å^2^)

**Table:**

	*x*	*y*	*z*	*U*_iso_*/*U*_eq_	
Cu1	0.15932 (5)	0.57426 (4)	1.06176 (4)	0.02211 (16)	
O1	0.2117 (3)	0.4082 (2)	0.9252 (2)	0.0217 (5)	
O2	−0.0365 (3)	0.2769 (2)	0.8582 (2)	0.0234 (5)	
O3	0.3034 (3)	0.5715 (3)	0.2622 (2)	0.0254 (6)	
O4	0.0605 (3)	0.4498 (2)	0.1767 (2)	0.0243 (5)	
N1	0.3031 (3)	0.7066 (3)	0.9800 (3)	0.0227 (6)	
N2	0.5273 (3)	0.9828 (3)	0.6389 (3)	0.0212 (6)	
C1	0.1118 (4)	0.3055 (3)	0.8481 (3)	0.0201 (7)	
C2	0.1732 (4)	0.2095 (4)	0.7247 (3)	0.0226 (7)	
H2A	0.283116	0.198415	0.750049	0.027*	
H2B	0.103647	0.112435	0.695960	0.027*	
C3	0.1715 (4)	0.2823 (4)	0.6072 (3)	0.0202 (7)	
C4	0.2935 (4)	0.3977 (4)	0.6063 (4)	0.0237 (8)	
H4	0.377601	0.430724	0.679879	0.028*	
C5	0.2926 (4)	0.4638 (4)	0.4992 (3)	0.0224 (7)	
H5	0.376877	0.541559	0.499491	0.027*	
C6	0.1706 (4)	0.4188 (3)	0.3911 (3)	0.0206 (7)	
C7	0.0445 (4)	0.3072 (4)	0.3944 (4)	0.0262 (8)	
H7	−0.042266	0.277462	0.323303	0.031*	
C8	0.0466 (4)	0.2403 (4)	0.5012 (4)	0.0255 (8)	
H8	−0.039000	0.164151	0.502236	0.031*	
C9	0.1809 (4)	0.4860 (4)	0.2720 (3)	0.0217 (7)	
C10	0.2529 (4)	0.8020 (4)	0.9196 (3)	0.0235 (7)	
H10	0.147482	0.819245	0.929866	0.028*	
C11	0.3479 (4)	0.8759 (4)	0.8433 (3)	0.0238 (8)	
H11	0.307795	0.943106	0.802968	0.029*	
C12	0.5025 (4)	0.8526 (4)	0.8249 (3)	0.0221 (7)	
C13	0.5560 (4)	0.7578 (4)	0.8919 (4)	0.0267 (8)	
H13	0.661744	0.740613	0.885116	0.032*	
C14	0.4550 (4)	0.6888 (4)	0.9684 (4)	0.0258 (8)	
H14	0.494412	0.625784	1.014908	0.031*	
C15	0.6109 (4)	0.9265 (4)	0.7382 (4)	0.0259 (8)	
H15A	0.688256	1.007259	0.801480	0.031*	
H15B	0.672420	0.856634	0.686588	0.031*	
C16	0.6446 (4)	1.0736 (3)	0.5796 (4)	0.0226 (7)	
H16A	0.719702	1.015295	0.532433	0.027*	
H16B	0.706851	1.153652	0.655442	0.027*	
C17	0.4372 (4)	0.8651 (4)	0.5234 (4)	0.0235 (7)	
H17A	0.356366	0.802431	0.560496	0.028*	
H17B	0.510889	0.805222	0.475975	0.028*	

### Atomic displacement parameters (Å^2^)

**Table:**

	*U*^11^	*U*^22^	*U*^33^	*U*^12^	*U*^13^	*U*^23^
Cu1	0.0226 (3)	0.0256 (3)	0.0190 (3)	0.00159 (17)	0.00451 (17)	0.00903 (18)
O1	0.0243 (12)	0.0234 (12)	0.0184 (12)	0.0049 (10)	0.0047 (10)	0.0067 (10)
O2	0.0245 (13)	0.0253 (12)	0.0206 (13)	0.0031 (10)	0.0067 (10)	0.0065 (10)
O3	0.0255 (13)	0.0294 (13)	0.0222 (13)	−0.0033 (11)	0.0018 (10)	0.0143 (11)
O4	0.0252 (13)	0.0309 (13)	0.0176 (13)	0.0001 (10)	0.0015 (10)	0.0115 (10)
N1	0.0235 (15)	0.0248 (15)	0.0201 (15)	0.0030 (12)	0.0032 (12)	0.0075 (12)
N2	0.0230 (15)	0.0207 (14)	0.0190 (15)	−0.0013 (12)	0.0029 (12)	0.0071 (12)
C1	0.0272 (19)	0.0233 (17)	0.0171 (17)	0.0093 (15)	0.0049 (14)	0.0150 (14)
C2	0.0251 (18)	0.0240 (17)	0.0215 (18)	0.0078 (14)	0.0044 (14)	0.0084 (14)
C3	0.0233 (17)	0.0249 (17)	0.0159 (17)	0.0085 (14)	0.0062 (14)	0.0082 (14)
C4	0.0243 (18)	0.0276 (18)	0.0171 (18)	0.0026 (14)	0.0002 (14)	0.0039 (14)
C5	0.0253 (18)	0.0216 (17)	0.0189 (18)	−0.0008 (14)	0.0025 (14)	0.0061 (14)
C6	0.0245 (18)	0.0239 (17)	0.0153 (17)	0.0071 (14)	0.0054 (14)	0.0063 (14)
C7	0.0232 (18)	0.037 (2)	0.0169 (18)	−0.0013 (15)	−0.0018 (14)	0.0102 (15)
C8	0.0216 (18)	0.0310 (19)	0.0233 (19)	−0.0038 (15)	0.0052 (15)	0.0117 (15)
C9	0.0272 (18)	0.0240 (17)	0.0147 (17)	0.0075 (15)	0.0056 (14)	0.0038 (14)
C10	0.0236 (18)	0.0286 (18)	0.0197 (18)	0.0052 (15)	0.0027 (14)	0.0087 (15)
C11	0.0317 (19)	0.0233 (17)	0.0185 (18)	0.0088 (15)	0.0015 (15)	0.0072 (14)
C12	0.0244 (18)	0.0225 (17)	0.0164 (17)	0.0001 (14)	0.0017 (14)	0.0027 (14)
C13	0.0221 (18)	0.0304 (19)	0.029 (2)	0.0052 (15)	0.0039 (15)	0.0104 (16)
C14	0.0266 (19)	0.0282 (19)	0.0251 (19)	0.0047 (15)	0.0011 (15)	0.0123 (15)
C15	0.0225 (18)	0.0299 (19)	0.0244 (19)	0.0007 (15)	0.0023 (15)	0.0086 (15)
C16	0.0223 (18)	0.0215 (17)	0.0219 (19)	−0.0030 (14)	0.0027 (14)	0.0070 (14)
C17	0.0251 (18)	0.0213 (17)	0.0228 (19)	−0.0014 (14)	0.0035 (14)	0.0071 (14)

### Geometric parameters (Å, º)

**Table:**

Cu1—Cu1^i^	2.8992 (8)	C5—H5	0.9500
Cu1—O1	1.969 (2)	C5—C6	1.388 (5)
Cu1—O2^i^	1.968 (2)	C6—C7	1.400 (5)
Cu1—O3^ii^	2.299 (2)	C6—C9	1.492 (5)
Cu1—O4^ii^	1.985 (2)	C7—H7	0.9500
Cu1—N1	1.979 (3)	C7—C8	1.381 (5)
Cu1—C9^ii^	2.466 (3)	C8—H8	0.9500
O1—C1	1.252 (4)	C10—H10	0.9500
O2—C1	1.264 (4)	C10—C11	1.375 (5)
O3—C9	1.244 (4)	C11—H11	0.9500
O4—C9	1.299 (4)	C11—C12	1.392 (5)
N1—C10	1.343 (4)	C12—C13	1.389 (5)
N1—C14	1.346 (4)	C12—C15	1.513 (5)
N2—C15	1.458 (4)	C13—H13	0.9500
N2—C16	1.467 (4)	C13—C14	1.380 (5)
N2—C17	1.473 (4)	C14—H14	0.9500
C1—C2	1.525 (4)	C15—H15A	0.9900
C2—H2A	0.9900	C15—H15B	0.9900
C2—H2B	0.9900	C16—H16A	0.9900
C2—C3	1.514 (5)	C16—H16B	0.9900
C3—C4	1.396 (5)	C16—C17^iii^	1.508 (5)
C3—C8	1.391 (5)	C17—H17A	0.9900
C4—H4	0.9500	C17—H17B	0.9900
C4—C5	1.378 (5)		
			
O1—Cu1—O3^ii^	101.92 (9)	C8—C7—H7	120.1
O1—Cu1—O4^ii^	92.30 (10)	C3—C8—H8	119.3
O1—Cu1—N1	89.47 (11)	C7—C8—C3	121.3 (3)
O2^i^—Cu1—O1	157.53 (10)	C7—C8—H8	119.3
O2^i^—Cu1—O3^ii^	99.04 (9)	O3—C9—Cu1^iv^	67.55 (18)
O2^i^—Cu1—O4^ii^	90.56 (10)	O3—C9—O4	120.8 (3)
O2^i^—Cu1—N1	93.02 (11)	O3—C9—C6	120.8 (3)
O4^ii^—Cu1—O3^ii^	61.65 (9)	O4—C9—Cu1^iv^	53.30 (16)
N1—Cu1—O3^ii^	104.62 (10)	O4—C9—C6	118.3 (3)
N1—Cu1—O4^ii^	166.22 (11)	C6—C9—Cu1^iv^	171.6 (3)
C1—O1—Cu1	125.2 (2)	N1—C10—H10	118.7
C1—O2—Cu1^i^	122.9 (2)	N1—C10—C11	122.6 (3)
C9—O3—Cu1^iv^	82.5 (2)	C11—C10—H10	118.7
C9—O4—Cu1^iv^	95.0 (2)	C10—C11—H11	119.8
C10—N1—Cu1	123.5 (2)	C10—C11—C12	120.3 (3)
C10—N1—C14	117.3 (3)	C12—C11—H11	119.8
C14—N1—Cu1	118.7 (2)	C11—C12—C15	122.9 (3)
C15—N2—C16	109.1 (3)	C13—C12—C11	116.8 (3)
C15—N2—C17	111.4 (3)	C13—C12—C15	120.3 (3)
C16—N2—C17	108.0 (3)	C12—C13—H13	120.1
O1—C1—O2	126.7 (3)	C14—C13—C12	119.8 (3)
O1—C1—C2	116.9 (3)	C14—C13—H13	120.1
O2—C1—C2	116.3 (3)	N1—C14—C13	123.0 (3)
C1—C2—H2A	110.3	N1—C14—H14	118.5
C1—C2—H2B	110.3	C13—C14—H14	118.5
H2A—C2—H2B	108.6	N2—C15—C12	114.2 (3)
C3—C2—C1	107.1 (3)	N2—C15—H15A	108.7
C3—C2—H2A	110.3	N2—C15—H15B	108.7
C3—C2—H2B	110.3	C12—C15—H15A	108.7
C4—C3—C2	120.2 (3)	C12—C15—H15B	108.7
C8—C3—C2	121.3 (3)	H15A—C15—H15B	107.6
C8—C3—C4	118.5 (3)	N2—C16—H16A	109.5
C3—C4—H4	119.8	N2—C16—H16B	109.5
C5—C4—C3	120.3 (3)	N2—C16—C17^iii^	110.8 (3)
C5—C4—H4	119.8	H16A—C16—H16B	108.1
C4—C5—H5	119.4	C17^iii^—C16—H16A	109.5
C4—C5—C6	121.2 (3)	C17^iii^—C16—H16B	109.5
C6—C5—H5	119.4	N2—C17—C16^iii^	110.2 (3)
C5—C6—C7	118.8 (3)	N2—C17—H17A	109.6
C5—C6—C9	119.3 (3)	N2—C17—H17B	109.6
C7—C6—C9	121.9 (3)	C16^iii^—C17—H17A	109.6
C6—C7—H7	120.1	C16^iii^—C17—H17B	109.6
C8—C7—C6	119.9 (3)	H17A—C17—H17B	108.1
			
Cu1—O1—C1—O2	−12.1 (5)	C5—C6—C9—O3	6.7 (5)
Cu1—O1—C1—C2	164.4 (2)	C5—C6—C9—O4	−175.2 (3)
Cu1^i^—O2—C1—O1	28.1 (4)	C6—C7—C8—C3	0.5 (5)
Cu1^i^—O2—C1—C2	−148.4 (2)	C7—C6—C9—O3	−170.6 (3)
Cu1^iv^—O3—C9—O4	1.2 (3)	C7—C6—C9—O4	7.4 (5)
Cu1^iv^—O3—C9—C6	179.2 (3)	C8—C3—C4—C5	−2.7 (5)
Cu1^iv^—O4—C9—O3	−1.4 (3)	C9—C6—C7—C8	174.8 (3)
Cu1^iv^—O4—C9—C6	−179.5 (2)	C10—N1—C14—C13	−3.5 (5)
Cu1—N1—C10—C11	−168.9 (2)	C10—C11—C12—C13	−2.8 (5)
Cu1—N1—C14—C13	168.5 (3)	C10—C11—C12—C15	177.8 (3)
O1—C1—C2—C3	−84.4 (3)	C11—C12—C13—C14	2.0 (5)
O2—C1—C2—C3	92.4 (3)	C11—C12—C15—N2	−20.0 (5)
N1—C10—C11—C12	0.5 (5)	C12—C13—C14—N1	1.2 (5)
C1—C2—C3—C4	79.3 (4)	C13—C12—C15—N2	160.6 (3)
C1—C2—C3—C8	−98.3 (4)	C14—N1—C10—C11	2.6 (5)
C2—C3—C4—C5	179.7 (3)	C15—N2—C16—C17^iii^	−179.7 (3)
C2—C3—C8—C7	179.7 (3)	C15—N2—C17—C16^iii^	−178.5 (3)
C3—C4—C5—C6	0.6 (5)	C15—C12—C13—C14	−178.7 (3)
C4—C3—C8—C7	2.1 (5)	C16—N2—C15—C12	169.9 (3)
C4—C5—C6—C7	2.0 (5)	C16—N2—C17—C16^iii^	−58.8 (4)
C4—C5—C6—C9	−175.4 (3)	C17—N2—C15—C12	−71.1 (4)
C5—C6—C7—C8	−2.6 (5)	C17—N2—C16—C17^iii^	59.2 (4)

Symmetry codes: (i) −*x*, −*y*+1, −*z*+2; (ii) *x*, *y*, *z*+1; (iii) −*x*+1, −*y*+2, −*z*+1; (iv) *x*, *y*, *z*−1.
